# Toscana virus – an emerging Mediterranean arbovirus transmitted by sand flies

**DOI:** 10.1099/jgv.0.002045

**Published:** 2024-11-07

**Authors:** Yonca Keskek Turk, Koray Ergunay, Alain Kohl, Joseph Hughes, Clive S. McKimmie

**Affiliations:** 1 School of Medicine, University of Leeds, Leeds, UK; 2 Walter Reed Biosystematics Unit (WRBU), Smithsonian Institution, Museum Support Center, Suitland, MD, USA; 3 One Health Branch, Walter Reed Army Institute of Research (WRAIR), Silver Spring, MD, USA; 4 Department of Entomology, Smithsonian Institution–National Museum of Natural History (NMNH), Washington, DC, USA; 5 Department of Medical Microbiology, Virology Unit, Faculty of Medicine, Hacettepe University, Ankara, Türkiye; 6 Centre for Neglected Tropical Diseases, Departments of Tropical Disease Biology and Vector Biology, Liverpool School of Tropical Medicine, Pembroke Place, Liverpool, UK; 7 MRC-University of Glasgow Centre for Virus Research, Glasgow G61 1QH, Scotland, UK; 8 Skin Research Centre, York Biomedical Research Institute, Hull York Medical School, University of York, York, UK

**Keywords:** arbovirus, diagnostics, epidemiology, pathogenesis, vector-borne disease

## Abstract

Toscana virus (TOSV) is an emerging arthropod-borne virus (arbovirus) of medical importance that is increasing its range across much of the Mediterranean Basin, Europe and the Middle East. Transmitted by *Phlebotomus* spp. sand flies, it is the most clinically relevant sand fly-borne phlebovirus. Initially isolated in the Tuscany region of Central Italy, it has now been detected in multiple countries that surround this geographical area. Infection of the vertebrate host can cause fever and neurological disease, following the dissemination of the virus to the brain. The prevalence is high in some regions, with a notable percentage of individuals showing seroconversion. TOSV can be a leading cause of acute meningitis and encephalitis (AME) during the summer months. In this comprehensive review, we will focus on several key topics. We discuss how TOSV has spread to establish outbreaks of infection in both humans and animals around the Mediterranean and the wider region. Clinical aspects of TOSV infection in humans are described, along with the best standards in diagnosis. Finally, we focus our discussion on the role of the sand fly vector, describing their biology, vector competency, implications for putative vertebrate reservoirs, the effect of the climate emergency on sand fly distribution and the putative role that sand fly-derived salivary factors may have on modulating host susceptibility to TOSV infection.

## Introduction

Toscana virus (TOSV) is a medically important virus belonging to the *Phlebovirus* of the *Phenuiviridae* family, in the recently classified *Hareavirales* order of the *Bunyaviricetes* class of negative-sense RNA viruses [1,2]. Transmitted by multiple species of sand flies, including several within the *Phlebotomus* genus, infection typically causes a febrile-like illness with occasional development of severe disease, following the dissemination of the virus to neural tissue. It was first isolated from sand flies in the Tuscany region of Central Italy in 1971 [3], and infection can cause fever followed by neurological disease as the virus disseminates to the brain [4,5], with TOSV being the leading cause of meningitis and encephalitis during the summer months in some regions [6]. Infection of multiple putative reservoir vertebrate species has been documented, including agriculturally important livestock, dogs, cats and bats. As such, it has a remarkable ability to persist within endemic areas, triggering sporadic outbreaks of infection in the warmer months, when its sand fly vector is more numerous. A combination of factors including the climate emergency and increasing globalization is expanding the range of both sand flies and TOSV to new geographic areas. Nonetheless, research on this agent has been neglected, with only a limited understanding of TOSV biology and the diseases that result from infection. The absence of licensed antivirals and vaccines indicates an urgent unmet need to better understand TOSV biology, pathogenesis and its ecology. In this review, we provide a comprehensive summary of the key findings relating to this increasingly important virus.

**Fig. 1.**
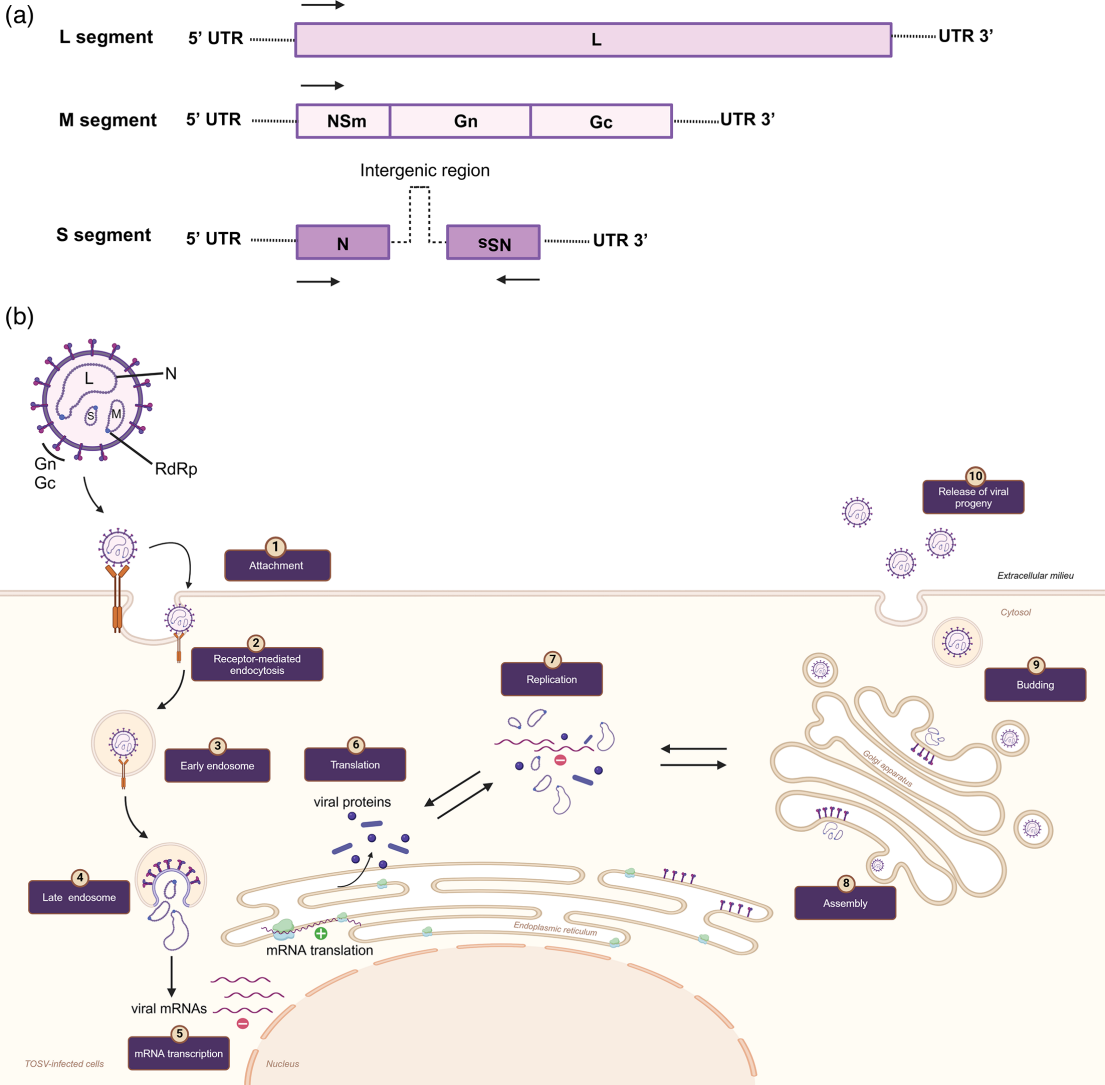
Genome organisation (a) and replication cycle (b) of TOSV. (**a**) TOSV contains a tripartite single-stranded RNA genome. L segment (negative-sense) encodes the viral component of the RNA-dependent RNA polymerase (RdRp). M segment (negative-sense) encodes Gn/Gc and a non-structural protein (NSm). S segment encodes (ambisense) the nucleocapsid (N) protein and a non-structural protein (NSs). (**b**) (1) Phleboviruses, including TOSV, use virus-encoded glycoproteins, Gn and Gc, to bind cell surface molecules DC-SIGN, L-SIGN and heparan sulphate (glycosaminoglycan). This is similar to the related phenuiviruses that also target DC-SIGN. Receptor use is likely cell specific, e.g., with DC-SIGN mediated entry occurring in dendritic cells (DCs). (2) Virus enters the cell via receptor-mediated endocytosis. (3) Once internalized, the viral particles move through early and late endosomes. (4) In late endosomes, acidification induces the membrane fusion activity of the Gc protein with the endosomal membrane. This fusion triggers the encapsidation of the viral genome, and virus RdRps are released into the cytoplasm, where primary transcription and replication occur. (5) N interactions with RdRp allow access to the ribonucleoprotein (RNP), which serves as a template for the transcription of new mRNA. Following the translation of the viral mRNAs and genome replication (6–7), viral Gn/Gc are cleaved by host cell proteases in the endoplasmic reticulum, allowing Gn–Gc glycoprotein heterodimers to reach the Golgi apparatus, (8) where viral assembly occurs. (9) Newly formed virions decorated with Gn and Gc in the Golgi bud via vesicles to the plasma membrane and (10) are released from the host cell by exocytosis. Replication within arthropod cells, although likely similar, is not well studied and requires definition. The figures were created on BioRender.com.

TOSV is a tri-segmented, negative-sense RNA with a diameter of ~100 nm [7,8]. Like all bunyaviruses, the genome consists of three segments called small (S), medium (M) and large (L), reflecting their nt length (summarized in Fig. 1a). The replication strategy of TOSV has mostly been inferred from the studies on other bunyaviruses (Fig. 1b) [8,13]. One recent study has defined TOSV’s entry mechanism into mammalian cells, demonstrating that it shares its acid-activated membrane fusion strategy with other Bunyavirales. Here, they found that TOSV cell entry interestingly relies on late endosomes, in the early stages of maturation, and thereby displayed greater resistance to endocytic degradation and flexibility in pH-dependent fusion [14]. However, vector receptors need to be defined for the virus entry mechanism. Similar to other *Bunyaviricetes*, the S segment encodes the nucleocapsid (N) protein responsible for encapsulating the viral RNA replication products to form the ribonucleoprotein complex (Fig. 1). The M segment encodes a polyprotein precursor, which then is cleaved into Gn and Gc components via host cell proteases in the endoplasmic reticulum. The Gn–Gc heterodimer is involved in virus assembly and attachment to new target cells. The L segment encodes the viral component of the RNA-dependent RNA polymerase (RdRp) [15]. The S and M segments also encode NSs and NSm, which are non-structural proteins, respectively [15,16]. Although NSs is not required for efficient *Phlebovirus* replication in cultured mammalian cells [e.g. Rift Valley fever virus (RVFV)] [15], NSs is required for efficient suppression of the IFN response [17,20] and therefore likely has an important role in suppressing anti-viral immunity during infection *in vivo*. The specific molecular aspects of TOSV replication and genetics are not well described compared to other *Bunyaviricetes*. In the absence of this, one can draw on findings from other genetically similar arboviruses, such as RVFV, an important human and animal pathogen [9,21], and the novel family of *Phenuiviridae* viruses (e.g. Uukuniemi uukuvirus). Together, these can serve as a guide for understanding TOSV structure and replication, which have been described elsewhere [14,22, 23].

Phylogenetic analyses have revealed three distinct lineages of TOSV, denoted as A, B and C. Presently, there are no differences in virulence or clinical symptoms among these genetic lineages. In some countries, such as France (lineages A and B), Türkiye (lineages A and B) and Croatia (lineages B and C), at least two lineages are known to coexist [6]. Whether these distinct lineages have different animal reservoirs requires further research. Recently, the complete sequence of the TOSV strain 1500590, a lineage A virus, was made available, leading to the establishment of the first reverse genetic system capable of recovering infectious recombinant TOSV (rTOSV) from cDNA. This advancement allows for the creation of genetically modified versions of the virus. By generating an NSs-deficient rTOSV capable of expressing reporter genes, it enables the visualization and tracking of intracellular replication, essential for further research and vaccine development efforts [24]. A developed reverse genetic system is also available for a lineage B strain of TOSV [17]. In comparison, research with lineage C virus is challenging, as it only exists as a sequence, although it could be possible for a reverse genetics strategy to rescue lineage C.

**Fig. 2.**
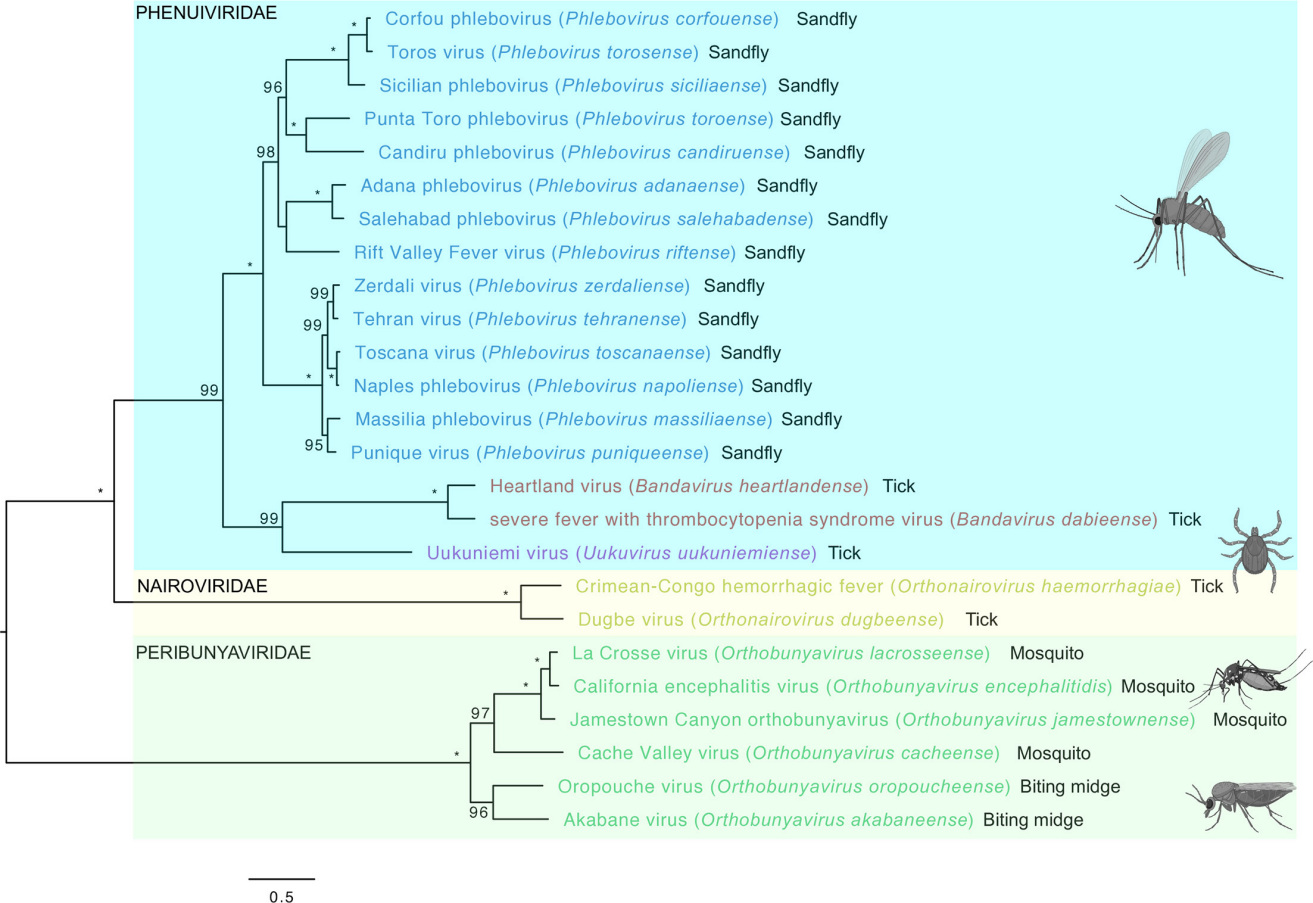
Phylogenetic tree of arboviruses of medical and veterinary importance. Maximum likelihood tree of the RdRp aligned using MAFFT and reconstructed using IQTREE2. Tips are annotated with the virus name, the species name in brackets and the arthropod vector. Values at the nodes represent the bootstrap support, only values above 70 are shown and 100 bootstrap support is represented with a *. The tree is mid-point rooted, the branch lengths are drawn to scale and the scale bar represents the number of amino acid changes per site.

To demonstrate the phylogeny of TOSV and related Bunyavirales, we have undertaken a *de novo* analysis of all *Bunyaviricetes* viruses by comparing RdRp sequences and plotting those viruses that are of medical importance to humans (Fig. 2). The main vector that transmits each virus is also annotated. This shows how TOSV and the other genetically related phleboviruses are all transmitted by sand fly vectors. The sequence similarity that these genetically related viruses have with other *Bunyaviricetes* viruses, which are transmitted by mosquito and tick vectors, is shown. This includes three other *Phenuiviridae* viruses that are transmitted by ticks. More distantly related are the *Nairoviridae* viruses, also transmitted by ticks, and the *Peribunyaviridae* viruses that are transmitted by either mosquito or midge vectors. This includes the Oropouche virus, which is an important emerging *Bunyaviricetes* virus responsible for ongoing outbreaks in South America.

## Epidemiology of TOSV: a historical context

Due to a combination of factors including the climate emergency, globalization, urbanization and changes in countryside management, the burden of arboviral diseases (including those caused by TOSV) on human health is increasing. TOSV cases in humans have been reported across the wider Mediterranean region and, as such, pose a risk of infection to millions living in these regions. Importantly, the number of TOSV cases is on the rise [25]. After its first identification from sand flies in Italy, in 1971, early seroepidemiological studies frequently did not assess TOSV distribution, often referred to as ‘*Phlebotomus* fever’ or ‘Pappataci fever’ [26,27]. It was not until 10 years after its identification that TOSV was registered in the International Catalogue of Arthropod-Borne Viruses [28]. TOSV is not alone, as *Phlebotomus* spp. sand flies can also transmit the genetically related *Phlebovirus napoliense* and *Phlebovirus siciliaense* viruses. Infection with these two viruses is limited to febrile illness, with no neurological involvement. Importantly, TOSV is the only sand fly-borne phlebovirus that can cause neurological disease [6]. TOSV has since been detected across a wide geographic range, including much of Southern Europe, Africa and the Mediterranean region. In the Mediterranean region, the majority of TOSV infections occur in the warmer months between May and October, peaking in August. This time frame corresponds to the peak activity of the sand fly vector [5]. In some of these regions, antibody prevalence in humans for sand fly-borne viruses is greater than 50% [27], suggesting that TOSV and related viruses have long imposed a substantial, underappreciated burden on human health. The ability of TOSV to be neurovirulent was first shown following the investigation of cerebrospinal fluid (CSF) from a Swedish patient with encephalitis, who had recently visited Portugal [4]. TOSV infection has also been reported in Swedish United Nations soldiers following stays in Cyprus [29,30]. Perhaps not surprisingly, there is a high antibody prevalence rate of 20% to TOSV among Cyprus’s local population [27]. Travellers returning from TOSV-endemic regions also regularly present with evidence of TOSV infection [31,33]. Table 1 describes a complete list of epidemiological studies evaluating TOSV burden in these regions. However, it is important to note that cross-reactions might have influenced the estimation of the seroprevalence rate. Serological cross-reactions have been documented within the sand fly fever Naples virus complex, of which TOSV is a member [34]. Indeed, our new phylogenetic comparison shows that TOSV is highly related to a number of viruses including Naples phlebovirus, to which it is most related, and the Zerdali, Tehran, Massilia and Punique phleboviruses (Fig. 2).

**Table 1.** Epidemiological studies of TOSV

Location	Year	Cases/findings	References
USA	1985, 2009, 2015	Three imported cases of TOSV meningitis or meningoencephalitis from Italy were reported	[31,52, 197]
UK	2019	One imported case was determined with TOSV encephalitis	[198]
Sweden	1980s	An imported TOSV case was reported from Swedish tourists visiting Spain	[199]
Southern Europe			
Southern Italy	2000s	Meningitis and encephalitis cases were reported during the summer	[200,202]
Southern France	2001, 2004	Two TOSV cases manifested as aseptic meningitis and influenza-like illnesses, while one case was diagnosed as acute meningitis	[203,204]
Catalonia, Spain	Early 2000s	A 6% seroprevalence of anti-TOSV IgG was found, with two acute clinical cases with viral meningitis or meningoencephalitis	[205]
Spanish Mediterranean and Madrid	Early 2000s	The overall seroprevalence was 24.9%, with higher rates observed in rural populations compared to urban areas	[73,206,208]
Emilia-Romagna and Umbria, Italy	2002, 2003	The first reports of CNS infections caused by TOSV were documented	[209,210]
France	2003, 2006	Imported acute meningitis and meningoencephalitis cases due to TOSV infection were reported	[211,212]
Portugal	2002–2005	TOSV meningitis was confirmed in 6 (5.6%) cases out of 106 samples tested	[213]
Portugal	2004–2008	The prevalence was 4.2% in those with neurological symptoms and 1.3% in those without neurological symptoms	[50]
Kosovo	2005	The presence of TOSV in the population was suggested	[214]
Bosnia and Herzegovina	2006–2008	Anti-TOSV IgG and IgM were analysed in 68 human serum samples, revealing recent infection in 7 patients (10.29%)	[215]
Croatia	2007–2009	TOSV seropositivity was 37.5% among healthy residents	[216]
Ionian Islands, Greece	2010	TOSV IgG antibody prevalence was 51.7% in Corfu and 39% in Cephalonia	[217]
Northern Italy	2010	Acute meningitis cases due to TOSV infection were reported	[218]
Greece	2010–2014	TOSV was responsible for 10% of CNS infections	[219]
Greece	2010–2014	Three different TOSV cases were reported, two of which showed neurological symptoms	[51,62, 220]
Tuscany, Italy	2012	A total seropositivity of 10% was recorded for TOSV	[221]
Sicily, Italy	2012	TOSV-specific IgG prevalence was 25% in those with neurological symptoms and 10.8% in those without neurological symptoms	[222,223]
Emilia-Romagna, Italy	2012	Among 120 suspected neuroinvasive infection cases, TOSV was detected in 28.3%. Of these, 79.4% were in the acute phase of infection	[224]
Aegean Sea Islands, Greece	2013	TOSV seroprevalence was 21%	[225]
Northern Greece	2013	TOSV seroprevalence was 11.26%	[226]
Corsica, France	2014	TOSV RNA was detected in *Phlebotomus* species sand flies	[227]
Portugal	2009–2018	Six TOSV cases were identified from patients who had CNS infections	[228]
Madrid, Spain	2007, 2018–2019	The seroprevalence was 34.5% overall, with anti-TOSV IgG at 41.5% in 2007 and 21.3% in 2018–2019	[229]
Elba Island, Italy	2018	Twelve cases of TOSV meningoencephalitis with symptoms were reported	[230]
Southern Tuscany, Italy	2011–2019	TOSV positivity was 4.6% in CSF samples, and TOSV-specific IgM was 27.1% in sera	[231]
Southwest Portugal	2019	Neutralizing antibodies to TOSV were found in 5.3% of healthy blood donors	[232]
Corsica, France	2019	TOSV antibodies were found in 22.5% of cases using virus microneutralization assay	[233]
Central Europe			
Germany	1993–1994	Thirteen acute TOSV infections were reported in German citizens returning from Southern France, Greece, Italy and Portugal	[33,234, 235]
Germany	1993–1995	Out of 317 patients, 13 (4.1%) tested positive for TOSV antibodies; these cases were imported from Italy (11 cases, 84.6%), Portugal (1 case, 7.7%) and Turkey (1 case, 7.7%)	[236]
Netherlands, Germany	2000s	Imported TOSV infectious-caused CNS diseases were reported	[237,238]
Switzerland	2008, 2009, 2012	Imported aseptic meningitis cases due to TOSV infection were reported from Swiss tourists who visited in Italy	[239,241]
Africa			
Tunisia	2003–2009	IgM (10%) and IgG (7%) antibodies for TOSV were identified in patients with neurological diseases	[242]
Djibouti, Africa	2010–2011	The circulation of Toscana-related viruses was 3.7%	[243]
Tunisia	2013	Anti-TOSV IgG positivity was 9.5% in healthy individuals	[244]
Tunisia	2013	TOSV-neutralizing antibodies were present in 41% of human sera, with confirmed co-circulation with Punique virus	[91,245]
Northern Algeria	2013	The presence of TOSV was confirmed in sand flies and the population with almost 50% seropositivity	[246]
Tunisia	2014	Anti-TOSV IgM was 12.16% in serum samples, TOSV positivity was 12.86% in CSF samples and TOSV RNA was found in pooled sand fly samples	[247]
Algeria	2016–2018	TOSV infection was found in 3.8% of patients with neurological symptoms	[248]
Libya	2013–2014	TOSV seroprevalence was 25% in the population	[249]
Middle East			
Türkiye	2010	Fourteen TOSV infection cases were reported in patients initially diagnosed with aseptic meningoencephalitis	[250]
Central, North and Southeast Anatolia, Türkiye	2011–2012	TOSV seroprevalence was 17.8% in asymptomatic blood donors and 15.7% in patients with CNS infections of unknown cause	[85,251]
Eastern Thrace, Türkiye	2012	TOSV seroprevalence in blood donors was 14.4%, and the first co-infections of WNV and TOSV were reported	[252]
Central Anatolia, Türkiye	2012	Among 94 patients investigated, TOSV seroreactivity was found in 37.2% (35 patients)	[77]
Mediterranean region, Türkiye	2011–2012	Neutralizing antibodies to TOSV were detected in 13.9% of healthy blood donors	[253]
Western border of Iran	2013	Among military personnel, 1% TOSV was revealed in serum samples	[254]
Türkiye	2014	A patient who was HIV positive was also found to have an acute TOSV infection	[255]
Western Saudi Arabia	2012–2016, 2019	The circulation of TOSV showed an overall seroprevalence of 0.8% in residents	[135]

The time frame indicates the years during which the samples were collected or when TOSV diagnoses were made in patients. CNS, central nervous system; HIV, Human Immunodeficiency virus; WNV, West Nile virus.

The clinical importance of TOSV infections is underlined by studies assessing the viral aetiology of central nervous system (CNS) infection among children in Tuscany, Italy. Importantly, these showed that TOSV infection is responsible for at least 80% of summertime viral infections of the CNS in children [35,36]. This peak of CNS infection was coincident with a high frequency of adult insect vectors (*Phlebotomus perniciosus* and *Phlebotomus perfiliewi*), which typically peaks in August [37]. In addition, the seroprevalence studies suggest that an L proportion of infection occurs without obvious clinical CNS involvement. For example, in nine different regions of Spain, the seroprevalence of TOSV in a random cohort of individuals was 26% (*n* = 1268 individuals) that had not presented with meningitis or febrile illness [38]. Here, although antibodies to TOSV were found in younger age groups, they were detected at a higher frequency in older age groups [39]. This has also been shown in an Italian cohort, with an age-dependent seroprevalence of TOSV, with 19.8% in adults and 5.8% in children [40]. Interestingly, those who have exposure to sand fly-enriched environments, such as forestry workers, demonstrate seroprevalence rates as high as 77.2% [41]. A more recent assessment of sand fly-transmitted virus in Italy over a 10-year period described TOSV seropositivity between 22.95 and 26.75% [42].

Further evidence for increased geographical dissemination has come from a recent prevalence study in Bulgaria, which found seropositivity at 24.4% [43]. In Southwest Germany, 4% of individuals with probable viral meningoencephalitis in a retrospective cohort analysis exhibited neuroinvasive TOSV, despite having no prior history of visiting an endemic region [44]. To assess the exposure to sand fly-borne infections in general, one study from Spain assessed seropositivity to sand fly salivary proteins. Here, the seroprevalence to *Phlebotomus perniciosus* sand fly salivary gland homogenate and recombinant protein rSP03B were investigated to detect sand fly exposure in blood donors, with seroprevalences estimated at 69 and 88%, respectively. The same study showed 26% of TOSV seropositivity in blood samples [45].

In summary, TOSV infection is now widespread across multiple countries. It is likely that further spread to more temperate countries will occur as the climate warms and international travel continues apace [46,47].

## Clinical manifestations of TOSV infection

Similar to many arboviruses, the majority of TOSV infections are either asymptomatic, mild or undiagnosed [48]; however, an increasing number of cases develop severe disease that can be life threatening or leave disabling sequelae, including those that involve deafness [49]. Typically, the infection manifests as a mild febrile illness, commonly marked by elevated body temperature, headaches, skin rashes (exanthema) with haemorrhagic features, feelings of sickness, muscle pain, joint pain, arthritis and occasionally nausea and vomiting [50,51]. Following a bite by an infected sand fly, the virus is assumed to replicate in the skin and then disseminates to the systemic circulation, at which point clinical signs can become apparent. This incubation period can range from 3 to 7 days, before the onset of more severe clinical signs. However, the incubation period of this infection can be considerable. In one case report, the incubation period extended to 17 days in a patient who developed extreme lethargy, malaise, anhedonia and decreased hearing [52], while another study assessing infection in a travel-acquired cohort estimated the incubation period at an average of 12 days [53]. During the early stage phase of infection that coincides with viraemia, TOSV RNA can also be detected in urine, a characteristic that is sometimes observed in virus infections that involve the nervous system [54,57].

### Complications of TOSV infection

Signs of meningitis, sudden hearing loss and other neurological involvement can develop in some cases 2 weeks post-fever. These are diverse and can manifest differently and can encompass Kernig’s sign (resistance to knee extension with hip flexed), stiffness in the neck, light sensitivity, tremors, nystagmus (involuntary eye movements), muscle weakness, double vision, sleep disturbances, prolonged fatigue, altered mental alertness and changes in consciousness. Typically, these symptoms can endure for several weeks, while persistent alterations in personality linked to TOSV encephalitis have also been reported [58,60], which can also occur without concurrent meningitis [61,62]. TOSV has the potential to cause fatal encephalitis in humans, although this is rare. In one fatal case, the diagnosis was based on positive serological results and the patient’s travel history to Tuscany prior to the development of symptoms, which indicated progressive encephalitis linked to TOSV infection [63]. Moreover, in Romania, severe encephalitis and meningoencephalitis caused by TOSV were identified through real-time reverse transcription polymerase chain (RT-PCR) testing, following five deaths out of eight patients [55]. More frequent is the presentation of TOSV-linked hydrocephalus, a complication of viral meningoencephalitis that is otherwise highly uncommon. Patients with CNS TOSV infection have also reported testicular pain including epididymal-orchitis, epididymitis and genital vasculitis [64,68] although no evidence yet suggests direct infection of testes [65]. Interestingly, TOSV RNA was detected in seminal fluid samples from a patient with TOSV meningitis [69], suggesting possible transmission through sexual intercourse.

Persistent sequalae of infection have also been noted. In one case, a patient presented with severe neurological features after returning from Umbria, Italy. Here, serum and CSF were positive for antibodies against TOSV, and recovery was associated with persistent headaches [70]. There have also been reports of siblings who contracted severe life-threatening meningoencephalitis following TOSV infection and have since experienced long-lasting neurological complications, including hydrocephalus [64].

TOSV infection associated with peripheral neuropathy has been reported to mimic a Guillain–Barré-like syndrome. Although one case report was insufficient to demonstrate a definitive association [71], in a further case-control study, TOSV was recognized as a contributing factor to the development of Guillain–Barré syndrome [72]. Due to the scarcity of cases, it is not clear whether these TOSV neurological manifestations of the peripheral nervous system can be considered an established feature of this disease. TOSV infection also has the capacity to rarely cause persistent neurological infections accompanied by ischaemic complications [73], sensory polymyeloradiculopathy [66] and brachial plexus involvement [74].

Complications arising from TOSV infections do not always involve CNS disease [75]; lymphadenopathy [76], pancytopenia during acute infection [77], benign myositis and fasciitis [78] have been described. The connection between myositis and viral infections is well established [79]. The mechanisms behind these viral-­induced pathologies remain unclear and warrant further research. In summary, TOSV infections are of growing clinical importance. In the absence of an animal model that recapitulates TOSV disease, the majority of our insights on TOSV pathogenesis have come from observations in the clinic. Understanding the molecular and cellular basis for these diverse pathologies, and the host response to infection, is important if we are to improve the care and treatment of these patients.

## Diagnosis of TOSV infection

The diagnosis of TOSV infection typically involves clinical evaluation and laboratory testing, emphasizing the assessment of symptoms, medical history and travel to TOSV-endemic areas. Given the rise in imported cases, especially in countries where the virus is not endemic, it is increasingly important for medical professionals to consider TOSV as a differential diagnosis, particularly in cases of AME in individuals who have recently returned from the Mediterranean region [52,80].

For patients with CNS involvement, differential diagnosis can be aided through the use of virological tests to confirm TOSV infection and so differentiate from, e.g. West Nile virus (WNV), enterovirus and herpesvirus infections among others. These assays are typically PCR based and can detect low concentrations of viral RNA. PCR testing detects viral genetic material and is considered indirect virological testing. PCR does not confirm active viral infection, as residual RNA can persist in some tissues for many months post-infection. Sensitivity is an important consideration for cases with neurological involvement, as the prior acute viraemic phase is rapid and infectious virus can be cleared from the blood, most likely through binding neutralizing antibodies, before clinical presentation [81]. To date, several PCR assays have been established to detect TOSV, including nested RT-PCR of serum and CSF samples, which are additionally employed for those with the acute meningitis [82,83]. Typically, assay primers amplify the TOSV S fragment [36]. More recently, real-time PCR assays have been adopted due to their time-saving benefits and reduced risk of contamination [84]. Although CSF sampling is most informative, recent developments have optimized the detection of TOSV RNA using real-time RT-PCR in more accessible blood samples from patients with CNS disease [85,87]. Lately, a TOSV real-time RT-PCR assay has been established to target three specific genomic regions within the nucleoprotein gene. This assay offers a robust and sensitive method for detecting TOSV by targeting multiple genomic regions, enhancing the specificity and reducing the risk of false negatives [88]. Additionally, diluted urine samples have been shown to be suitable for TOSV RNA detection using this Trio TOSV real-time RT-PCR system [89]. Finally, there now exists a multiplex PCR where several sets of primers are employed in a single reaction to identify infections caused by either TOSV or enterovirus [81].

Serological assays, such as ELISA and immunofluorescence assay, can be used to detect current and previous infections. As such, they are less informative for diagnosis but may offer the only sign of TOSV infection if the acute viraemia has passed and TOSV RNA can no longer be detected. These assays detect specific antibodies (IgM and IgG) against TOSV in the patient’s blood serum. There is limited cross-reactivity among viruses belonging to the *Phlebovirus* genus, particularly between the TOSV and *Phlebovirus napoliense*, mainly because of the significant similarity in the N protein of these viruses [90]. As such, these tests are of most use when screening many specimens rapidly, e.g. for seroprevalence studies. Notably, the neutralization assay has a lower likelihood of cross-reactions compared to indirect immunofluorescence assay or ELISA [38,91]. Several commercial assays are available for the detection of TOSV antibodies, providing diagnostic options for healthcare professionals when assessing potential TOSV infections [92].

Alternatively, diagnosis can be based on isolating the virus. Here, clinical samples, especially CSF, are used to infect cells (e.g. Vero or BHK-21) and monitored for detection of cytopathic effects in cells. This approach is more powerful when combined with sequencing to define the aetiological agent. However, in practice, this is rarely undertaken due to its complexity and the availability of other diagnostic techniques. In addition, there are concerns that these cell culture-based approaches, which, e.g. monitor cytopathic effect, are not sufficiently sensitive compared to PCR [93]. For example, a recent study that incorporated metagenomic next-generation sequencing has been used as a differential diagnostic tool in undiagnosed meningitis cases and revealed 8 cases (8/23) caused by TOSV [94]. Much insight could be generated if sequencing of the full infectious genome was undertaken, especially alongside epidemiological studies. This could help define a number of key TOSV infection attributes such as mutation rates, strain selection and whether specific strains of TOSV are more likely to involve CNS tissue.

In summary, prompt diagnosis allows for appropriate medical care and management of patients with TOSV infections [95]. To provide rapid diagnosis and minimize cross-reactivity, it is important that the clinician chooses the gold standard for TOSV detection, which can incorporate a combination of specific serology and PCR-based approaches. Although there are no specific treatments for TOSV infection, appropriate clinical management can improve outcomes, while a more widespread use of clinical TOSV detection would enable a more accurate definition of TOSV prevalence.

## *Phlebotomus* spp. (Diptera: *Psychodidae*) sand fly: vectors of TOSV

### Transmission features

Sand flies belong in the order Diptera, suborder Nematocera, family *Psychodidae* and subfamily *Phlebotominae*. Six primary sand fly genera are recognized, three of which are found in the Old World (*Phlebotomus*, 13 subgenera; *Sergentomyia*, 10 subgenera; and *Chinius*, 4 species) and three of which are found in the New World (*Lutzomyia* 26 subgenera and groups; *Brumptomyia,* 24 species; and *Warileya*, 6 species) [96]. Notably, the genera *Lutzomyia* and *Phlebotomus* and some *Sergentomyia* [97] are those that are anthropophilic and exhibit competence to transmit pathogens [98].

Geographically, sand flies are present between 50° N and 40° S latitudes, but are absent in New Zealand and the Pacific islands [98]. Phlebotomine sand flies principally exist in the warmer climates of Asia, Africa, Australia, Southern Europe and the Americas [99]. Importantly, it is predicted that this range will extend to new transmission zones because of climate change [100]. In addition to *Phleboviruses,* phlebotomine sand flies are also responsible for the spread of *Leishmania* (*Leishmania*) spp. (Kinetoplastida: *Trypanosomatidae*) and *Bartonella bacilliformis* [101,102]. Of the 900 sand fly species, less than 100 can transmit *Leishmania* parasites, while just nine species of sand flies transmit *Phleboviruses*, including TOSV [93,101, 102]. *Phlebotomus perniciosus* and *Phlebotomus perfiliewi* have been identified as vectors of TOSV [103,104]. Although not yet documented, it is likely that other related species such as *Phlebotomus sergenti*, *Phlebotomus longicuspis*, *Phlebotomus neglectus*, *Phlebotomus tobbi* and *Sergentomyia minuta* could also participate in TOSV transmission [105,107].

The contributing factors for TOSV maintenance in nature are not well known. Interestingly, both male and female sand flies have been identified with TOSV infection. As in other haematophagous insects, only females acquire blood meals; therefore, infection in male sand flies suggests vertical and/or transovarial transmission [104]. Indeed, experimentally infected sand fly species, including *Phlebotomus perniciosus*, can transmit TOSV transovarially [108,110]. In addition, transovarially infected female sand flies can transmit TOSV by biting a susceptible vertebrate [110]. Experimental evidence further suggests venereal infection in female sand flies, which might serve as an infection amplifier in the absence of other reservoirs [111]. However, the presence of vertebrate reservoirs is likely to be required for TOSV maintenance, as viral infection rates in sand fly colonies not exposed to viraemic vertebrates steadily drop with each succeeding generation of the colony [110,112, 113].

The capacity of TOSV to circulate horizontally among members of the same generation has also been suggested based on work on the related phlebovirus Massilia virus [114,115]. Interestingly, infection of sand flies was more efficient if included with a sugar meal. This could suggest that virus deposited by sand flies, as they seek nectar, may be an efficient method to infect other sand flies that feed from the same site [116].

### Potential vertebrate reservoirs of TOSV

With the high prevalence of TOSV seroconversion in humans, the presence of a non-human vertebrate reservoir may not necessarily be a prerequisite. Nonetheless, numerous species of vertebrates have been proposed as TOSV reservoirs, although firm proof is still lacking. Temperature, humidity and airflow all have an impact on feeding activity. Though biting might occur indoors in darkened areas or among shaded vegetation during the day, most species feed around sunset and night when the temperature drops and humidity rises [99]. Adult sand flies can be found in caves and rock crevices, tree trunks or tree hollows, domestic animal enclosures, masonry crevices and other dark, humid locations such as basements and wells [117]. The vast range of vertebrate hosts that female sand flies feed on includes humans and various animals including canines, rodents, reptiles, amphibians and birds [101]. *Phlebotomus perniciosus* have a particularly varied feeding habit and typically are opportunistic feeders, biting whichever animal happens to be nearby [118,121].

Given the high frequency of either TOSV RNA detection or neutralizing antibodies found in canine blood samples taken during the sand fly season in Mediterranean Anatolia, Türkiye [121,122]; Portugal [123]; Corsica [124]; and Algeria [125,126], dogs have been proposed as a reservoir host. Indeed, the seroprevalence of TOSV was 6.8% in dogs and 3.7% in cats in the Portuguese study. Another seroprevalence study showed that guard dogs’ seroprevalence rate was 7.5% in two different regions of Tunisia [127], while there was 8.4% seropositivity in dogs from Greece [128]. Antibodies in cats to *Phlebotomus perniciosus* saliva (47.7%, 350/167) and neutralizing antibodies against TOSV (4.9%, 18/365) show that cats are bitten by sand flies and can be infected with TOSV [129]. However, it is not clear if either species supports the TOSV transmission cycle, due to the low level of viraemia that results from infection and inability to excrete virus [130]. However, experimental infection of dogs by *Phlebotomus perniciosus* feeding has been recently documented in a natural setting [131]. It has also been suggested that *Leishmania infantum*-infected canines demonstrate enhanced vectorial capacity for TOSV [132,133], compared to healthy dogs that do not have *L. infantum*. Livestock are also frequently bitten by sand flies, particularly *Phlebotomus perniciosus* [121]. These animals may also act as a reservoir, with 5–8% of serum samples exhibiting seropositivity (Kosovo, *n* = 1086 [134] and Saudi Arabia [135]). TOSV RNA is typically not detected in most cases, complicating host range definition [136], although infections of sheep and goat have also been suggested.

For several neurotropic arboviruses, such as WNV, birds have a role as viral amplification hosts, vector dispersion vehicles and sources of new strains by interspecies transmission. Birds passing through known migratory routes in the Hatay Province of Türkiye have been shown to be positive for TOSV RNA in samples taken from the brain and kidney [137]. In addition, the migratory common quail (*Coturnix coturnix*) exhibits a high seroprevalence rate of 42.45% in Spain [138]. Furthermore, a recent study conducted in the northern wetlands of Türkiye revealed the presence of both TOSV RNA and infectious virus in bird populations [139]. These findings indicate that birds may be a reservoir or act as an amplifying host for TOSV.

Bats have been recognized as important reservoirs of many zoonotic viruses worldwide [140]. However, there is little information about the role of bats in the ecology of *Phleboviruses*, including TOSV. While TOSV was once isolated from a bat’s brain (*Pipistrellus kuhlii*) [104], TOSV exposure rate can be considered low in bats as long-lived animals, with an antibody seroprevalence at 10%. Therefore, bat colonies are not likely to play a reservoir role for TOSV [141].

### Effect of the climate emergency on sand fly distribution

Zika, dengue and chikungunya viruses are well-described examples of agents that are transmitted by mosquitoes whose distribution has spread to over 130 countries [142]. As climate change becomes more pronounced, it is anticipated that, in addition to mosquitoes, other haematophagous insects like sand flies will also undergo geographical expansion. Climate has multiple impacts on the dynamics and prevalence of arthropod-borne infections. Invertebrate vectors, including sand flies, are ectothermic, and changes in the environment due to climate change can impact life cycle, movement, feeding activity and survival [143,144]. Many sand fly species have already been established across the Mediterranean region. Models predicted that just a 1 °C increase could create optimal environmental conditions for certain sand fly species (*Phlebotomus mascittii* and *Phlebotomus neglectus*) [145]. Subsequently, *Phlebotomus (Transphlebotomus) mascittii* was newly documented in Austria and across the Western Europe [146], while *Phlebotomus mascittii* has now been confirmed in central Europe, north of the Alps, France, Switzerland, Belgium and Germany [147]. As such, the TOSV vectors *Phlebotomus mascittii* and *Phlebotomus perniciosus* are now not only confined to Southern Europe but also identified in Germany [148], where autochthonous cases of TOSV meningoencephalitis have been reported [44,149]. In addition, changes in *Phlebotomus ariasi*, *Phlebotomus neglectus* and *Phlebotomus perfiliewi* distribution are being observed, with these species spotted in northern regions and higher altitudes, a shift attributed to climate change. The occurrence of imported TOSV infections in these locations poses a risk for potential local outbreaks if the competent vector species become established, as observed with other diseases transmitted by vectors. In summary, TOSV could extend its activity to new temperate regions where suitable vector species exist [150,153].

### TOSV and *Sergentomyia* sp.

Sand flies belonging to the genus *Sergentomyia* feed on a wide range of animals including reptiles, birds and a diverse array of mammals, occasionally including humans [154,155]. Some of these flies, including *S. minuta*, have been found positive for both human and *Leishmania* DNA [120] and can transmit *Leishmania* to reptiles [96] and possibly to humans [156,159]. Importantly, this species may also transmit TOSV to vertebrates as they have been found harbouring TOSV RNA [160]. Furthermore, the *Phlebotomus*vectored Chandipura virus (CHPV) has also been detected in *Sergentomyia* sand flies from India. CHPV is a member of *Rhabdoviridae* that can, like TOSV, cause encephalitis [161]. Together, this suggests that *S. minuta* may also be competent for transmitting *Phleboviruses*, including TOSV [162].

### TOSV and *Lutzomyia* sp.

A pressing concern is the possibility that *Lutzomyia* sp. sand flies could transmit TOSV. This New World sand fly genus is found across South, Central and North America and consists of over 400 species, including *Lutzomyia (Lu.) longipalpis* (Diptera: *Psychodidae: Phlebotominae*). This species is an important vector of several medically important pathogens, including *Leishmania* and potentially also arboviruses [96]. Indeed, it is the main vector of *L. infantum* in the Americas that causes visceral leishmaniasis. *Lu. longipalpis* has broad-range feeding habits and has different habitats, including rural and urban areas feeding on humans, pets, livestock, rodents, bats and opossums [163,165]. However, studies investigating the potential capability of this key vector to become infected and transmit arbovirus are urgently required [166].

While laboratory-based infection of *Lu. longipalpis* with *Phlebotomus*-transmitted *Phlebovirus siciliaense* and *Phlebovirus napoliense* is inefficient [167], there is evidence to suggest competence as an arboviral vector. For example, Punta Toro virus (PTV, also a phlebovirus) is transmitted by *Lutzomyia* species in Panama [168], while the Candiru complex viruses (family *Phenuiviridae*) have been isolated from *Lutzomyia* species, some of which cause febrile illness in humans [169]. Viola phlebovirus, a putative new viral species and a novel *Phlebotomus* fever serogroup member, was identified in *Lu. longipalpis* species in Brazil. The ability of this new sand fly-derived virus to replicate within mammalian cell lines and express NSs and NSm proteins suggests that the virus may be a novel arbovirus [170].

A *Lu. longipalpis* cell line can be infected and replicate a wide range of arboviruses, although PTV infection was inefficient [171,172]. This is surprising as PTV has been isolated from both humans and sand flies and may suggest that the cell line may be derived from a cell type resistant to infection. Interestingly, the replication of Bunyavirales in *Lu. longipalpis* is nonetheless possible as RVFV can replicate following the intrathoracic inoculation (albeit not via blood feeding) and be transmitted to RVFV-susceptible mammalian hosts [173]. Despite RVFV’s ability to infect multiple species, this is intriguing considering the geographic separation of vector (Americas) with RVFV (Old World) and their lack of co-evolution to date [173]. Interestingly, *Lu. longipalpis* may mechanically transmit RVFV to other mammalian hosts after exposure to a virus donor blood meal [174]. The epidemiological significance of mechanical transmission of arboviruses needs to be clarified, as this transmission route could enable infection from vectors that are otherwise not considered competent vectors [174].

To date, with the geographic range of *Lu. longipalpis* and TOSV not overlapping, it is not surprising that there is no field evidence that this sand fly can be infected or transmit TOSV to vertebrate hosts. There may also be sequence adaptions required by TOSV for infection of *Lutzomyia* species for transmission to be sufficiently efficient. Nonetheless, in our globalized world of international travel, there is a risk of TOSV viraemic individuals becoming exposed to biting sand flies of the Americas [175].

## Putative role of sand fly saliva in modulating TOSV infection

Saliva deposited by biting haematophagous arthropods is biologically active in vertebrates. Like all arbovirus life cycles, sand fly-vectored TOSV involves continual transfer between vertebrate hosts and vectors. Following a blood meal from an infected vertebrate host, the virus undergoes replication within the sand fly’s midgut. Subsequently, it migrates to the salivary glands, where it can be transmitted to a new host when the sand fly feeds on another blood meal. The transmission occurs when a female sand fly (males do not bite) inserts its mouthparts into the host’s skin, during which saliva and virus are deposited into the dermis [176].

Female sand fly saliva contains a blend of diverse pharmacologically active substances that have evolved to facilitate efficient feeding, including compounds with anti-haemostatic, vasoactive, immunomodulatory and anti-inflammatory properties [177]. These counteract vertebrate processes to enable efficient feeding but also have unintended consequences for mammalian susceptibility to pathogens including *Leishmania* infection. *Leishmania major*, the causative agent of cutaneous leishmaniasis, co-inoculated with salivary gland lysate from *Lu. longipalpis*, causes larger lesion size in the skin and higher parasite burden [178]. Indeed, compared to needle inoculation, *Leishmania* infection by sand fly bite increased the replication of this parasite in mice by many orders of magnitude and more severe disease. This is due to sand fly saliva/biting causing a rapid influx of neutrophils and monocytes, both which can become infected and support enhanced Leishmania infection [179,181].

Notably, mosquito saliva also plays an important role in determining the severity of arbovirus infections [182]. Factors in *Aedes* mosquito saliva enhance infection with a wide number of arboviruses including Bunyavirales RVFV, Cache Valley virus and Bunyamwera virus, resulting in increased mortality rates of mice [183,185]. In addition, the flavivirus dengue virus and alphaviruses (Semliki Forest virus and Chikungunya virus) infection are also enhanced by the presence of mosquito saliva in the inoculum. Similarly, WNV mixed with its vector (*Culex* mosquito) salivary factors caused higher viraemia, faster dissemination of the virus to tissues and earlier microinvasion compared to inoculation with WNV alone [186]. Besides mosquito-derived factors, tick saliva co-inoculated with Powassan virus (an encephalitic tick-borne flavivirus) also increases viral loads and alters the course of disease in mice, compared to mice infected with POWV alone [187].

It is not yet known whether sand fly saliva has a role in modulating bunyavirus infection, including TOSV infection, of the vertebrate host. Importantly, the lack of suitable immunocompetent mouse models of TOSV for investigating skin infection has hindered this, although a mouse model using a neuro-adapted strain of TOSV, with limited viral dissemination, has been defined [188]. The majority of salivary components of sand flies remain only partially characterized, and their specific roles are still unknown. Nonetheless, some interesting insights have been obtained, including salivary proteins such as antigen 5-related proteins, apyrases, odorant-binding proteins (including D7-related and PpSP15-like proteins), yellow-related proteins, silk-related proteins and lufaxin-like proteins [189]. The characterization of these salivary molecules and their biological activities have been discussed elsewhere [190,191].

Sand fly saliva is also immunomodulatory, which may alter host susceptibility to TOSV. Both *Lutzomyia* and *Phlebotomus* species’ saliva has an inhibitory impact on the activation of T cells, while promoting the expression of Th2-type cytokines [192,193]. Whether T cell modulation by saliva in the skin occurs sufficiently quickly to alter infection with the rapidly replicating TOSV is not known. Sand fly saliva is also highly inflammatory, inducing chemokine CCL2 expression and the recruitment of macrophages [194] and the expression of pro-inflammatory cytokines TNF-α, IL-6, CXCL8 and IL-12 [195]. Interestingly, saliva also has an impact on dendritic cells, stimulating the expression of IL-10 and prostaglandin E2, while decreasing the expression of co-stimulatory molecules Cluster of Differentiation 86 and also Major Histocompatibility Complex Class II may suppress this cell’s antigen-presenting function [196]. Considering that saliva has multiple effects on host physiology and immunity, it will be crucial to define whether sand fly saliva might also only influence the vertebrate host’s susceptibility to TOSV infection.

In conclusion, TOSV is an important and yet poorly understood cause of infectious neurological disease, especially in children. The high levels of prevalence in some endemic regions suggest that it also constitutes a substantial burden to non-neurological health, e.g. febrile illness. For those infections that cause neurological disease, the more routine inclusion of TOSV as a differential diagnosis, combined with more accurate molecular and/or serological testing will improve our estimate of the true burden of health imposed by TOSV. For those infections that do spread to neural tissue, our understanding of TOSV pathogenesis has almost exclusively been informed by observations made in the clinic. The development of an animal model that recapitulates aspects of human disease is therefore urgently needed to aid both our understanding of TOSV disease and the development of novel therapeutics. This is key, as numbers of TOSV cases are predicted to increase in the coming years, including in more temperate regions, in which the majority of individuals are immunologically naïve to TOSV. As such there is a clear unmet need to undertake more TOSV research.

## Abbreviations

AME: acute meningitis and encephalitis
CHPV: Chandipura virus
CHPV: Chandipura virus
CNS: central nervous system
CSF: cerebrospinal fluid
L: large
M: medium
N: nucleocapsid
PTV: Punta Toro virus
RdRp: RNA-dependent RNA polymerase
rTOSV: recombinant TOSV
RVFV: Rift Valley fever virus
S: small
TOSV: Toscana virus
WNV: West Nile virus
